# In these difficult times of COVID-19, urologic research cannot stop: COVID-19 pandemic and reconstructive urology highlighted in International Brazilian Journal of Urology

**DOI:** 10.1590/S1677-5538.IBJU.2020.04.01

**Published:** 2020-05-20

**Authors:** Luciano A. Favorito

**Affiliations:** 1 Unidade de Pesquisa Urogenital Universidade do Estado de Rio de Janeiro Rio de JaneiroRJ Brasil Unidade de Pesquisa Urogenital - Universidade do Estado de Rio de Janeiro - Uerj, Rio de Janeiro , RJ , Brasil ,; 2 Serviço de Urologia Hospital Federal da Lagoa Rio de JaneiroRJ Brasil Serviço de Urologia , Hospital Federal da Lagoa , Rio de Janeiro , RJ , Brasil

In times of great difficult because the Covid-19 infection the urologic research cannot stop. The July-August number of *Int Braz J Urol* , the fourth under my supervision, presents original contributions with a lot of interesting papers in different fields: Prostate Cancer, Uretral Stricture, Sexual Function, Male Incontinence, Buried Penis, Vesicoureteral Reflux, Prostate Biopsy, Kidney Transplant, Renal Cell Carcinoma, Bladder Cancer, BPH, Laparoscopy and Testicular Cancer. The papers came from many different countries such as Brazil, USA, Turkey, China, Korea, Coloumbia, Poland, Germany, Taiwan, India and Italy, and as usual the editor´s comment highlights some of them.

In the present issue we present three important reviews. Dr. Barbagli and colleagues from Italy performed in **page 511** ( 1 ) a nice narrative review about the bulbar urethral stricture treatment. This study is on the cover in this number. Dr. Barbagli is one of the most important urethral surgeons in the world and in this paper he present some tips and tricks developed along their prolonged surgical experience on the treatment of bulbar urethral strictures. Dr. Burks and colleagues from USA ( 2 ) present in **page 519** an important review about acquired buried penis (AABP). Dr. Burks shows that the management of AABP requires a combination of genitourinary reconstructive techniques and plastic surgery techniques that are unique to this condition and shows important tips and tricks for the treatment of AABP. Dr. Netto and collegues from Brazil presented in **page 523** ( 3 ) a nice paper about a consensus with practical orientation on how to evaluate and treat Vesicoureteral reflux in Brazil and addressed important recommendations on up to date choice of diagnosis evaluation and therapies. The editor in chief would like to highlight the following works too:

Dr. Porcaro and collegues from USA ( 4 ) on **page 545** evaluate the association between prostate volume index (PVI), and prostatic chronic inflammation (PCI) as predictors of prostate cancer (PCA). PVI is the ratio between the central transition zone volume (CTZV) and the peripheral zone volume (PZV) and concluded that high PVI and the presence of PCI lowered the mean rate of NPC and is associated with less aggressive tumor biology expressed by low tumor burden. PVI can give prognostic information before planning baseline random biopsies.

Dr. Demirtas and Collegues ( 5 ) from Turkey perfomed on **page 557** a interesting study about fusion prostate biopsy (FPB) and compare the pain levels in TRUS-guided standard 12-core prostate biopsy (SPB) and MpMRI-guided FPB. The authors concluded that FPB, with a relatively higher cancer detection rate, leads to the same pain level as SPB although it increases the number of biopsy cores and involves a more complex procedure compared to SPB.

Dr. Sefik and Collegues ( 6 ) from Turkey performed on **page 566** an interesting study about the preoperative renal function on survival outcomes in patients who underwent radical cystectomy (RC) with non-continent urinary diversion (UD) and concluded that overall mortality was higher and overall survival was lower in patients with preoperative eGFR <60mL/s. More patients had preoperative hydronephrosis with eGFR< 60mL/s.

Dr. Sipal and Collegues ( 7 ) from Turkey studied on **page 575** the reasons of storage symptoms (SS) after transurethral resection of the prostate (TURP) and they studied if was a positive correlation between preoperative and postoperative SS in patients with undergoing TURP and starting early solifenacin treatment in patients with high preoperative SS would be reasonable and concluded that TURP provides significant improvement in both storage and voiding symptoms. The predictive value of the preoperative S-IPSS on postop SS is significant. These results suggest that 5 mg solifenacin succinate treatment in the early postoperative period may be beneficial for patients with high preoperative SS and may not be beneficial in others. Small prostatic volume may bode ill for postoperative SS in the patients with de novo SS.

Dr. Liu and Collegues ( 8 ) from China shows on **page 585** explore the prognostic value of obesity (measured by BMI) on Renal Cell Carcinoma (RCC) in a systemic inflammation state and concluded that in localized RCC patients, obesity was an independent protective factor for cancer specific survival and recurrence free survival in a systemic inflammation state.

Dr. Vidal and Collegues ( 9 ) from Colombia studied on **page 599** evaluated the role of baseline clinical variables associated with overall survival (OS) and toxicity of Radium 223 (223Ra) and concluded that 223Ra therapy require an adequate selection of patients to obtain the greatest clinical benefit. Low basal Hb, hight basal alkaline phosphatase ALP and bone marrow involvement were the main factors that decreased overall survival in this study. 223Ra should be considered relatively early in the course of treatment.

Dr. Kretschemer and collegues ( 10 ) from Germany shows on **page 632** a interesting study about the effect of perioperative complications involving artificial urinary sphincter (AUS) implantation on rates of explantation and continence as well as health-related quality of life (HRQOL) and concluded that postoperative infections adversely affect device survival after AUS implantation and the comparative long-term functional results and HRQOL outcomes are similar between patients with or without perioperative complications.

In this very difficult times of COVID-19 I ask everyone to read the amazing editorial of Dr. Francisco Sampaio ( 11 ) our Emeritus Editor. Dr. Sampaio in his editorial show us important aspects of the Pandemic and in this issue he have one of the most complete reports on the clinical practice of Urology in COVID-19 Pandemic times in the briliant paper by Dr. Carneiro and Collegues on page 501 ( 12 ). We hope that readers will enjoy the present number of the *International Brazilian Journal of Urology.*
